# Evaluation of Respiratory Sounds Using Image-Based Approaches for Health Measurement Applications

**DOI:** 10.1109/OJEMB.2022.3202435

**Published:** 2022-09-13

**Authors:** Madison Cohen-McFarlane, Pengcheng Xi, Bruce Wallace, Karim Habashy, Saiful Huq, Rafik Goubran, Frank Knoefel

**Affiliations:** AGE-WELL NCECarleton University 6339 Ottawa ON K1S 5B6 Canada; AGE-WELL SAM3 National Innovation HubCarleton University 6339 Ottawa ON K1S 5B7 Canada; Digital Technologies Research CentreNational Research Council Canada 6356 Ottawa ON K1A 0R6 Canada; AGE-WELL SAM3 National Innovation HubCarleton University 6339 Ottawa ON K1S 5B7 Canada; AGE-WELL NCECarleton University 6339 Ottawa ON K1S 5B7 Canada; Bruyère Research Institute 152971 Ottawa ON K1N 5C8 Canada; National Research Council Canada 6356 Ottawa ON K1A 0R6 Canada; Department of Systems and Computer Engineering, Carleton University 6339 Ottawa ON K1S 5B6 Canada; Bruyère Research Institute, Bruyère Continuing CareElisabeth Bruyère Hospital 142139 Ottawa ON K1N 5C8 Canada; AGE-WELL NCECarleton University 6339 Ottawa ON K1S 5B6 Canada; AGE-WELL SAM3 National Innovation Hub Ottawa ON K1S 5B7 Canada

**Keywords:** Acoustic signal processing, audio visualization, convolutional neural network, cough classification, respiratory classification

## Abstract

*Goal:* The evaluation of respiratory events using audio sensing in an at-home setting can be indicative of worsening health conditions. This paper investigates the use of image-based transfer learning applied to five audio visualizations to evaluate three classification tasks (C1: wet vs. dry vs. whooping cough vs. restricted breathing; C2: wet vs. dry cough; C3: cough vs. restricted breathing). *Methods:* The five visualizations (linear spectrogram, logarithmic spectrogram, Mel-spectrogram, wavelet scalograms, and aggregate images) are applied to a pre-trained AlexNet image classifier for all tasks. *Results:* The aggregate image-based classifier achieved the highest overall performance across all tasks with C1, C2, and C3 having testing accuracies of 0.88, 0.88, and 0.91 respectively. However, the Mel-spectrogram method had the highest testing accuracy (0.94) for C2. *Conclusions:* The classification of respiratory events using aggregate image inputs to transfer learning approaches may help healthcare professionals by providing information that would otherwise be unavailable to them.

## I. Introduction

Cough is one of the most common, detectable and earliest respiratory symptom of many diseases. Currently respiratory assessments are done by a physician, typically during an in-person appointment by asking the patient to cough and breathe. In order to evaluate long-term variations in cough, the physician will typically ask the patient and/or family to describe their cough and how often it occurs. The reliability of subjective personal reporting can be inconsistent and becomes more unreliable when working with individuals who have co-morbidities that impact knowledge translation to the physician [1]. Physicians also listen for abnormal respiratory sounds using a stethoscope placed in specific locations on the chest and back [2]. The use of stethoscopes is outside the scope of this work; however, restricted breathing (RB) (wheeze or stridor) can be audible without amplification. Wheeze and stridor sounds are caused by an airway obstruction during the expiration and inspiration phase respectively [2]. These types of assessments are even more difficult when preformed remotely (via phone or video call) [3].

Respiratory related monitoring has been investigated previously as it relates to sleep (e.g., apneic periods) [4] and respiratory events [5], [6], [7], [8], [9]. A subset of this research focuses on audio-specific evaluations of cough in terms of identification, counting and classification [10], [11], [12], [13], [14], [15], [16]. The integration of this type of respiratory monitoring system has applications in existing assisted living systems aimed to support older adults living independently [17], [18]. Additionally, audio-based activities of daily living (ADL) have also been investigated to perform event recognition in the home [19], [20], [21].

Compared to the classification of images (computer vision), the classification of audio (computer hearing) is less studied as audio data are more scarce, though more datasets are currently being developed (e.g., Audio Set [22]). Additionally, when creating health-related classifiers, there are very few audio datasets that include detailed labels and those that do are very small. Respiratory sounds that are available publicly are typically cough sounds that are a class within a larger dataset (e.g., Audio Set [22]); however, no clinical information is provided. There are some cough/respiration specific datasets available, but they are much smaller. Pramono et al. compiled a small dataset of whooping coughs from YouTube [10], which was used to create a pertussis (whooping cough) classifier with an overall accuracy of 100% and zero false diagnosis [10].

In response to the global COVID-19 pandemic, there has been a growing number of publicly available databases of COVID-19 respiratory sounds available [23], [24], [25], [26], [27], [28]. This has led to more interest in the evaluation of cough as an indication of disease state [24], [28], [29], [30], [31], [32], [33], [34]. One area of interest is the transition of the COVID-19 cough from a dry cough, in early stages of the disease, to more wet-like cough in severe cases where the disease directly effects the lungs [35], [36], [37]. We have previously investigated a feature based differentiation between wet cough and dry cough [38]. This method was applied to the COVID-19 cough sounds in NoCoCoDa and showed that 77% of the COVID-19 cough sounds were more wet-like in nature [24].

Deep learning in computer vision has achieved great success in recent years. A series of deep Convolutional Neural Networks (CNN), including AlexNet [39] and VGG-19 [40], have achieved human-level performance in image classification benchmark tasks. In audio signal analysis, transfer learning has been applied to adapting these models for audio classification using audio visualizations. In order to use these methods, the one-dimensional audio signal must first be converted to a two-dimensional image-like representation. The most common audio visualization is the spectrogram, which has been used as the input to a variety of audio classification tasks [16], [41], [42], [43], [44], [45], [46]. In this paper, we focus on the application of transfer learning to classify respiratory sound characteristics, building on our previous work [16].

The identification of these events may help in the evaluation of underlying conditions (e.g., virus, bacteria, acute/chronic condition) during diagnosis [24]. A finalized version of this system may also be integrated into existing smart-home systems that support the independent living of older adults and the prevention of long-term hospitalizations by initiating early intervention [47]. Other applications include disease monitoring and contact tracing of respiratory conditions if the final system is integrated in large areas of high traffic [24].

## II. Materials and Methods

### A. Data Acquisition

The data used in this work were obtained from two main sources. The wet cough (wC) and dry cough (dC) sounds were obtained from Chatrzarrin et al. as they contained physician labeled events [38]. The whooping cough (whC) and restricted breathing (RB) events were obtained from links provided by Pramono et al., which were manually segmented and labeled (whC or RB) by a trained technician who was familiar with respiratory sound analysis [10].

Class distribution is presented in Table I. It was noted that there was a large class imbalance in this dataset. To address this issue, two additional datasets (undersampled and oversampled) were created and compared to determine the most appropriate data sampling method.

**TABLE I:** Dataset Distribution for the Original Dataset, the Undersampled Dataset and the Oversampled Dataset

Class	Number of Samples		
	Original Dataset	Undersampled Dataset	Oversampled Dataset
Wet Cough	27	19	97
Dry Cough	19	19	97
Whooping Cough	97	19	97
Restricted Breathing	25	19	97

### B. Preprocessing

Each sample was first downsampled to a sampling rate of 20 kHz. A lowpass filter was then applied to remove any high frequency noise (${f}_c = 4$ kHz), which was chosen based on the typical frequency range of cough sounds [48] .

### C. Audio Visualization

Previously we have investigated sound classifications using linear spectrograms [16]. Here we introduce three new visualization methods: linear spectrograms, logarithmic spectrograms, Mel-spectrograms, and wavelet scalograms. All visualizations were generated in MATLAB as described in Table II.

**TABLE II:** Audio Visualization Generation Parameters

Class	Parameters
Linear Spectrogram	‘spectrogram’ function with 90% overlap
Logarithmic Spectrogram	‘spectrogram’ function with 0.01 second Hann filter with 90% overlap and the y-scale set to logarithmic
Mel-Spectrogram	‘melSpectrogram’ function with a 0.01 second Hamming window with 90% overlap
Wavelet Scalogram	‘cwt’ function with a Morse wavelet

**Fig. 1.**
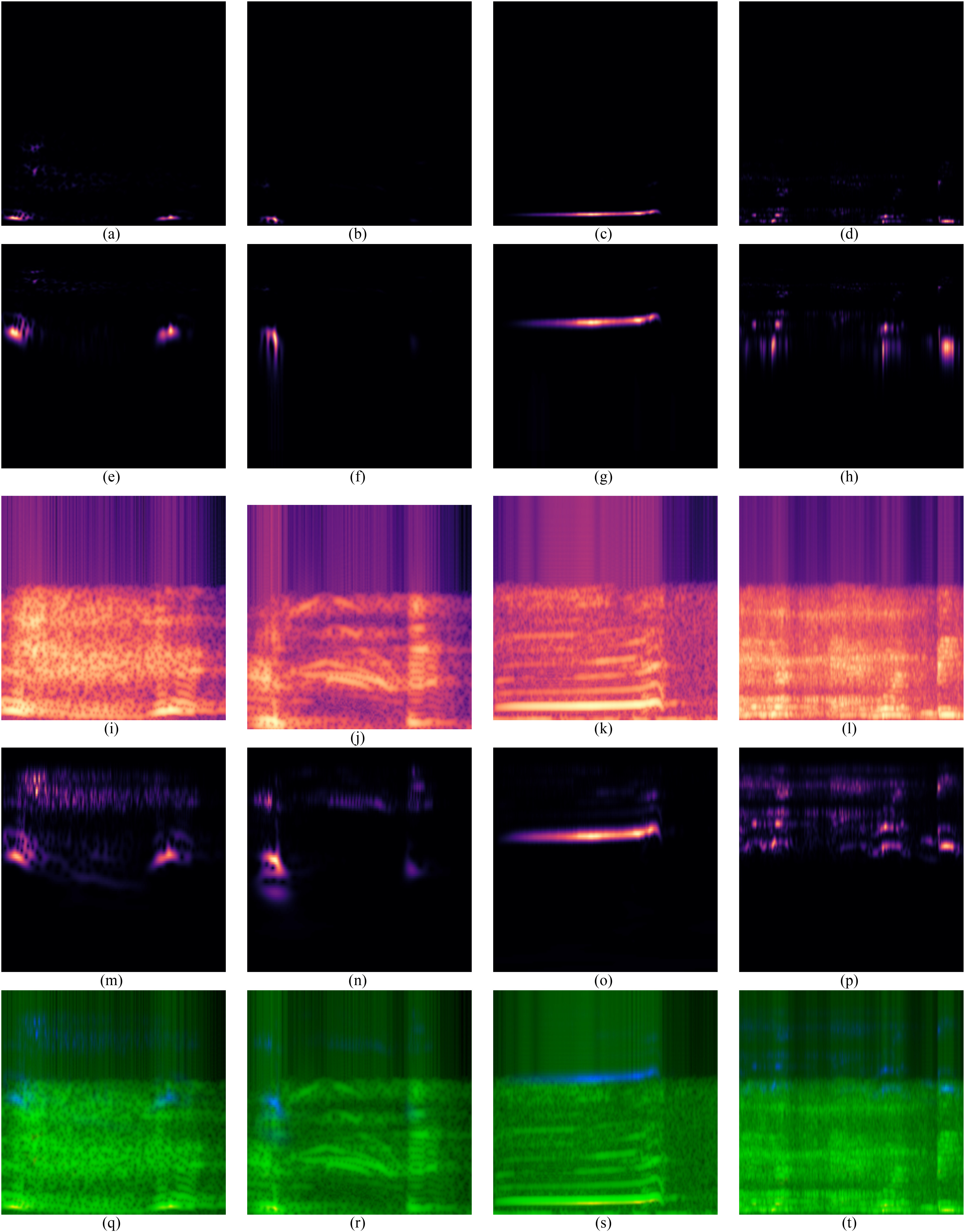
Respiratory sounds of a sample of each class using each visualization method. Linear spectrogram of (a) wC, (b) dC, (c) whC, and (d) RB; Logarithmic spectrogram of (e) wC, (f) dC, (g) whC, and (h) RB; Mel-spectrogram of (i) wC, (j) dC, (k) whC, and (l) RB; Wavelet scalogram of (m) wC, (n) dC, (o) whC, and (p) RB; Aggregate image visualization (linear spectrogram, Mel-spectrogram, and wavelet scalogram) of (q) wC, (r) dC, (s) whC, and (t) RB.

A sample linear spectrogram of each class is presented in Fig. 1(a)–(d). It can be seen that the majority of respiratory sounds occur at lower frequencies. To highlight the lower frequency ranges, logarithmic spectrograms were generated for all sounds, a sample of which is presented in Fig. 1(e)–(h).

Mel-spectrograms were also generated as they contain wider bands of lower frequency and narrower bands of high frequency information based on how the human ear interprets sound (Fig. 1(i)–(l)). When comparing these to the spectrogram visualizations, it is clear that more information is being captured, especially in the dC and whC classes where harmonics are visible. Finally, wavelet scalograms were generated using the continuous wavelet transform, which bins frequencies with respect to their expected duration (Fig. 1(m)–(p)). When comparing the wavelet scalograms to the Mel-spectrograms, there appears to be some variation, which may lead to deviations in classifier performance.

After each visualization was generated, they were adjusted to match the expected input size for the AlexNet classifier (227 x 227 x 3 pixels) [39]. As the classifier expects an RGB image, meaning that each image has three channels, each visualization was triplicated and stacked.

### D. Experimental Setup

Three classification tasks were considered (Table III). The tasks were identified based on expected classification challenges. To our knowledge, this work is one of the first to classify between wC, dC, whC, and RB (C1). The classification between wC and dC (C2) has been investigated in [38]. The final task in this work is the differentiation between cough sounds and RB sounds (C3) as one may be more interested in observing breathing sounds or cough sounds alone, especially from a clinical perspective.

**TABLE III:** Classification Tasks Evaluated Using All Four Audio Visualization Methods

Name	Task
C1	Wet cough (wC) vs. Dry cough (dC) vs. Whooping cough (whC) vs. Restricted Breathing (RB)
C2	wC vs. dC
C3	Cough vs. RB

The training procedure was the same for all classification tasks and was based on the procedure originally presented in [16]. All visualizations were grouped based on their respiratory class. Additionally, 90% overlap was chosen for the generation of the spectrogram visualizations. This was chosen in order to increase the pixel density in order to create visualizations that would most closely mirror the standard images expected by the classifier. To evaluate the impact of overlap choice, two classifiers were generated using linear spectrograms with 90% and 50% overlap. It was observed that the classification results are very similar, as described in the supplementary materials. Given the similarity, 90% overlap was chosen for all further evaluations as it creates visualizations that appear to have higher resolution, more closely mimicking standard images.

It was also observed that the oversampled dataset had the best performance when compared to the unequal and undersampled datasets. A detailed description of the data sampling evaluation based on linear spectrograms is included in the supplementary materials.

Oversampling was applied to all events by image duplication with a noise overlay. To ensure that the testing data only used held out independent data, eight samples (approximately 10% of the largest class size) from each class were set aside prior to data augmentation. The remaining samples from each class (wC, n = 19; dC, n = 11; whC, n = 89; RB, n = 17) were randomly duplicated to match the largest data class (whC, n = 89) and a noise overlay was applied on each duplicated image. The training data was further divided into 70% training and 30% validation during classifier training.

The AlexNet was adapted with weights pre-trained on the ImageNet dataset by removing the fully connected layers, appending new fully connected layers, and fine tuning the whole network for the respiratory event classification tasks. In order to retain the transferred features, a lower weight was assigned to the transferred convolutional layers and a higher weight was assigned to the newly added fully connected layers for fine tuning the model. All classifiers used stochastic gradient descent with momentum. The minimum batch size and maximum number of epochs were chosen based on trial and error (mini batch size of 15 with a maximum of 10 epochs). For all tasks, the initial learning rate was set to 1.0e-4 and data was shuffled for every new epoch. The third last layer was set as a fully connected layer with the adjusted number of classes (4 for C1 and 2 for C2 and C3), of which the learning rate is adjusted to 20 for faster learning. The second last layer was set to a soft max layer and the final layer was set as the classification layer based on cross entropy loss.

### E. Statistical Evaluation

For each classifier, four statistical metrics were calculated from validation (accuracy) and testing (accuracy, F1-score, and Cohen's Kappa Coefficient) in order to evaluate which paradigm performed best for each classification task. All results are summarized in Tables IV and V for each visualization method.

**TABLE IV:** Linear Spectrogram, Logarithmic Spectrogram, Mel-Spectrogram, and Wavelet Scalogram Classification Results When Used as the Input to the AlexNet Transfer Learning Paradigm for the Oversampled Dataset

Visualization Method	Task	Validation	Testing		
		Accuracy	Accuracy	F1-Score	Cohen's Kappa
*Linear Spectrogram*	C1	0.97	0.78	0.78	0.71
	C2	1.00	0.81	0.81	0.63
	C3	0.97	0.91	0.86	0.71
*Logarithmic Spectrogram*	C1	0.95	0.75	0.75	0.67
	C2	1.00	0.81	0.81	0.63
	C3	0.97	0.88	0.79	0.60
*Mel-spectrogram*	C1	0.97	0.84	0.84	0.79
	C2	1.00	0.94	0.94	0.88
	C3	0.99	0.88	0.79	0.60
*Wavelet Scalogram*	C1	0.97	0.88	0.87	0.83
	C2	1.00	0.81	0.81	0.63
	C3	0.98	0.88	0.79	0.60

**TABLE V:** Aggregate Visualization Classification Results When Used as the Input to the AlexNet Transfer Learning Paradigm for the Oversampled Dataset and the Results of the Additional Samples (Test2)

Vis. Method	Task	Val.	Test			Test2
		Acc.	Acc.	F1-Score	Cohen's Kappa	Acc.
*Aggregate*	C1	0.97	0.88	0.87	0.83	0.75
	C2	1.00	0.88	0.87	0.75	0.64
	C3	1.00	0.91	0.86	0.71	0.94

## III. Results

Table IV presents the results of all classification tasks for all visualization methods from the oversampled dataset. Cohen's Kappa Coefficient is a better indication of overall performance for C3 as the cough class (combination of wC, dC, and whC) contained 262 samples and the RB class consisted of 97 samples. Performance for the C1 and C3 tasks were lower in the logarithmic spectrogram-based method compared to the linear spectrogram-based method. However, the performance for C2 (wC vs. dC) was maintained across all measures between the linear and logarithmic spectrogram-based methods.

The performance of the Mel-spectrogram based classifiers is the highest for C2 (wC vs. dC) with a testing accuracy of 0.94. The Mel-spectrogram method also maintains high performance in C1 with a testing accuracy of 0.84. However, it had more difficulty differentiating between cough sounds and RB periods (C3), with a Cohen's Kappa Coefficient of 0.60.

The performance of the wavelet scalogram-based method for C2 (wC vs. dC) matches the performance of the linear and logarithmic spectrograms (Table IV), all of which are lower than the performance of C2 based on Mel-spectrograms. When looking at C1 (wC vs. dC vs. whC vs. RB), the wavelet scalogram-based method has the highest performance with a testing accuracy of 0.88. This method was also able to match the performance of both the logarithmic spectrogram and the Mel-spectrogram methods on C3 with a Cohen's Kappa Coefficient of 0.60, which is not as high as the linear spectrogram method (0.71).

Given the varied performance of each task across all visualization methods, an aggregate image-based method was proposed in order to create a single visualization method that would perform well across all tasks. Furthermore, 14 additional samples per class (Test2) have been obtained from publicly available sources and have been used to test the aggregate image approach. Table V presents the classification of the additional data. As expected, performance decreased on the new data. It was noted that the most confusion for C1 was related to 7/14 dry cough sounds being identified as wet cough sounds (a very difficult classification problem), which was also observed in C2. However, when testing the new data on C3, the performance increases to an accuracy of 0.94, supporting this method as a means of respiratory differentiation.

### A. Aggregate Image Generation and Evaluation

Since the highest performance for each task was not achieved using the same visualization method, an aggregate visualization is thus presented as a means to achieve high performance on all tasks. As shown in the previous section, when considering C1, the highest performance occurs with the wavelet scalogram. When considering C2, the Mel-spectrogram method had the highest performance. Finally, in C3 the linear spectrogram-based method had the highest performance. Therefore, the Mel-spectrogram, wavelet scalogram, and linear spectrogram visualizations were selected for the three RGB ‘like’ channels of the aggregate image.

**Fig. 2.**
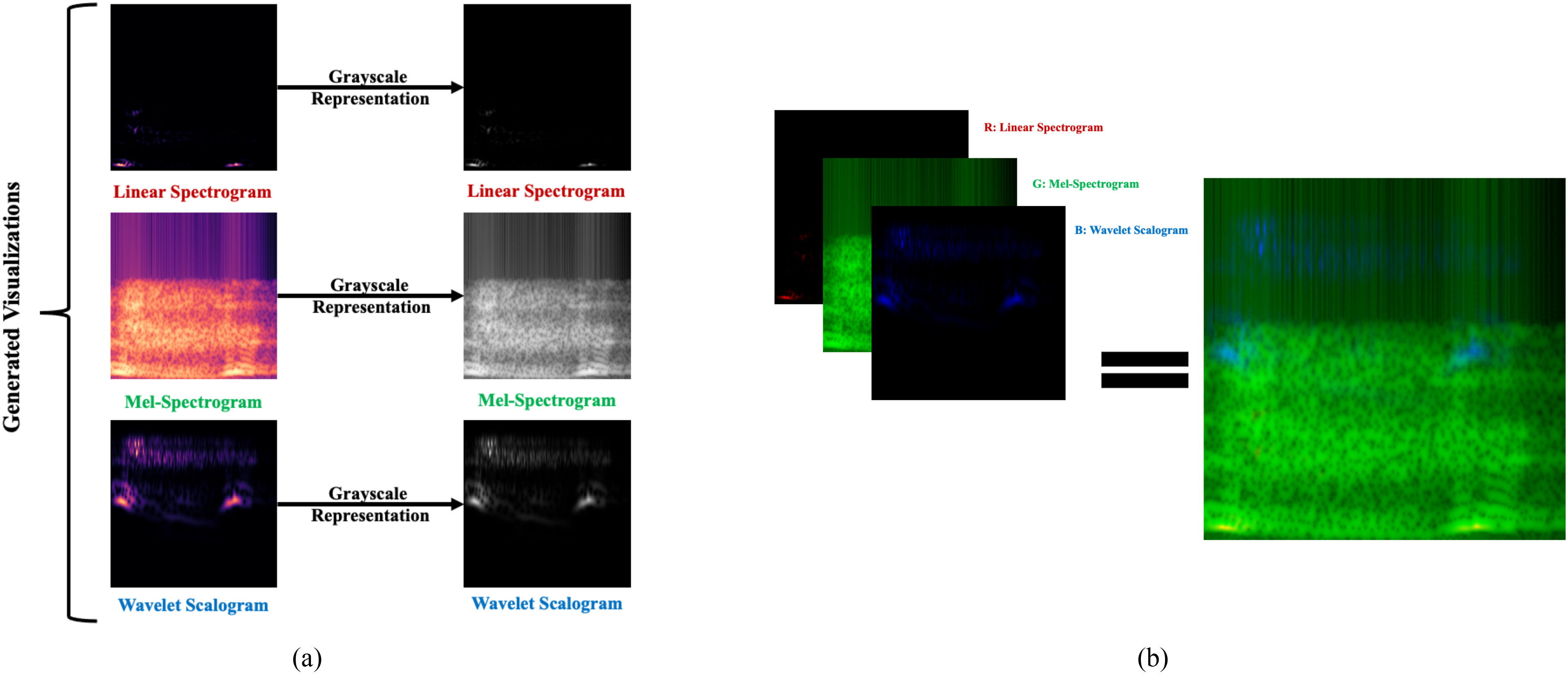
(a) Aggregate visualization generation of a wet cough using the grayscale representation of the linear spectrogram, Mel-spectrogram, and wavelet scalogram visualizations (b) Grayscale linear spectrogram assigned to the red channel, greyscale Mel-spectrogram assigned to the green channel, and wavelet scalogram assigned to the blue channel.

Aggregate images were created by first converting the chosen visualizations to grayscale for single channel representation (227 x 277 pixels), as shown in Fig. 2(a). Each grayscale visualization was then assigned to an RGB channel arbitrarily (Red = linear spectrogram, Green = Mel-spectrogram, and Blue = wavelet scalogram) and stacked to create a single aggregate visualization as shown in Fig. 2(b). Aggregate images of the previously separated training and testing sets were derived from the oversampled dataset and applied to each classification task using the previously described transfer learning procedure. Aggregate image representations of each class are also presented in Fig. 1(q)–(t).

Table V summarizes the aggregate image results and shows that this method was able to achieve the highest testing accuracy (0.88) for C1 (wC vs. dC vs. whC vs. RB). The aggregate-based method was able to achieve the second highest performance to the Mel-spectrogram method for C2 (wC vs. dC) with testing accuracies of 0.88 and 0.94 respectively. Finally, the aggregate image method led to the highest testing accuracy (0.91), matching the highest testing performance exhibited by the linear spectrograms for C3 (cough vs. RB). In summary, the aggregate visualization method was able to maintain the performance of C1 and C3 at a slight cost to the performance of the C2, meaning that the aggregate image-based method led to the best overall performance across all tasks.

In addition to the approach presented in this work, we also implemented a state-of-the-art deep learning model for audio data analysis called Self-Supervised Audio Spectrogram Transformer (SSAST). The SSAST is a deep neural network purely based on the self-attention mechanism for learning [49]. With a transformer-based backbone, similar to that of the vision transformer (ViT), the encoder outputs are fed into a joint objective function consisting of a generative and discriminative masked spectrogram patch modelling task [49]. The losses are computed using InfoNCE for the discriminative classification task and MSE for the reconstruction generative task [49]. Pre-training is undergone using the AudioSet and Librispeech datasets without labels [22], [50], and fine tuning is conducted on the four-class tasks C1. The SSAST model performed well with an accuracy and weighted F1-score of 0.83, the second highest overall performance on C1.

## IV. Discussion

This paper investigates the feasibility of classifying respiratory sounds using a visualization-based transfer learning approach. The respiratory sounds of interest included wC, dC, whC, and RB. Five visualization methods were presented in response to the idea that respiratory audio information may not be captured sufficiently by the linear spectrograms [16]. In addition to linear spectrograms, logarithmic spectrograms, Mel-spectrograms, wavelet scalograms, and aggregate images were generated for all respiratory sounds. The analysis focused on determining which combination of visualization method and classification task led to the highest performance.

When looking at the respiratory sounds in Fig. 1, some visual differences are present, which may be leveraged by the classifiers. When differentiating between the wC and dC (C2), the Mel-spectrogram (Fig. 1(i)–(l)) and wavelet scalogram (Fig. 1(m)–(p)) visualizations show that the wC has a more sustained response over the entire period compared to the dC, which presents with a gap between the first and second cough sound [38]. There is a clear continuous response exhibited in the whooping cough visualizations (Fig. 1(c), (g), (k), (o), (s)), which is different from the other events. Finally, when looking at the RB visualizations of the Mel-spectrograms (Fig. 1(i) & (l)), there appear to be more similarities between the wC and the RB. This may explain the lower performance of the Mel-spectrogram classifier for C1 (wC vs. dC vs. whC vs. RB), when compared to the highest performance for this task using the wavelet scalograms.

In C2 (wC vs. dC), the Mel-spectrogram method had the highest performance with a testing accuracy of 0.94. This is supported when looking at the visual differences between wC and dC in Fig. 1(i) and (j). Finally, the third task (C3, cough vs. RB) obtained the best performance using the linear spectrogram method with a Cohen's Kappa Coefficient 0.71.

As the best visualization method varied depending on the classification task, an aggregate image-based method was proposed and presented in Fig. 1(q)–(t). This method was able to match the highest individual performance of the C1 and C3 tasks with Cohen's Kappa Coefficients of 0.83 and 0.71 respectively (Table V). The aggregate image-based approach was able to outperform all other methods, with a testing accuracy of 0.88, except for C2. Therefore, the aggregate image-based method had the best overall performance across all tasks.

We note that if one is only interested in the differentiation of wC and dC sounds, the Mel-spectrogram based classifier would not only provide the best performance, but also reduce processing time as the aggregate image would not need to be generated. The classification of cough and RB has applications in cough monitoring, sleep disordered breathing, and remote monitoring of restricted breathing events. Here we have shown that these events are differentiable with a Cohen's Kappa Coefficient of 0.71 when using the aggregate image-based visualization.

The aggregate image channels were randomly assigned. This led to visualizations with very bright green channels (Fig. 1(q)–(t)), as the Mel-spectrograms were assigned to the second channel and contained high power compared to the other visualizations. Future work may include an iterative approach to the channel assignments in order to evaluate if certain combinations are more successful.

Finally, there are some difficulties associated with the deployment of these approaches. First of all, these methods rely on visualizations, which inherently increase computational load, inference time and may introduce a delay in processing when compared to classical audio processing approaches. We note that the performance of these methods were very similar when evaluating visualizations using a 50% overlap (described in the supplementary materials), meaning that reducing the overlap used in the visualization generation may be a means to reduce the computational load and inference time of this method.

Future work will include the deployment of these methods in a real-time, which will be applied to both the aggregate transfer learning approach and an optimized implementation of the SSAST across all tasks. Due to the scarcity of well labeled data for these classification tasks, we also acknowledge that the number of recordings used to develop the models was limited, especially for the dry and wet cough sounds. Additionally, the recordings used in this work were ideal in nature with very little background noise (though noise was added when applying the oversampling technique). All of these issues would need to be addressed to assess the long-term feasibility of this respiratory classification approach.

## V. Conclusion

Image classifiers based on deep neural nets have had great success in image recognition. Utilizing these existing classifiers to classify audio events facilitates feature extraction and reduces processing time when compared to classifiers built from scratch. In this paper we applied this idea to the classification of respiratory sounds. We evaluated three classification tasks (C1: wC vs. dC vs. whC vs. RB; C2: wC vs. dC; C3: cough (wC + dC + whC) vs. RB). An independent sub dataset was set aside for testing and the rest were oversampled for training and validating the models.

Classification performance varied depending on the task and visualization method used. The Mel-spectrogram and aggregate image methods had the highest performances when classifying between wC and dC (C2), with testing accuracies of 0.94 and 0.88 respectively. The aggregate image-based method had the highest performance when classifying between the four classes (C1) and between cough and RB (C3), with testing accuracies of 0.88 and 0.91 respectively. Furthermore, 14 of the samples not included in the original dataset were obtained for each class from public sources and have been used to evaluate the final aggregate image classifier. On the new data all classifiers struggled to differentiate between wet and dry cough sounds, a notoriously difficult problem. However, the aggregate method was able to achieve an accuracy 0.75 for the four-class problem (C1). Future work may include a deeper investigation into the hidden layers of the chosen method to fine-tune the model, increase the performance, and evaluate if there is unique involvement for specific respiratory events with a focus on the differentiation between wet and dry cough sounds,

Given the limited set of original recordings used in this work and the difficulty obtaining additional data, reported findings should be verified with a larger dataset. Specifically, future work will include the collection of more data across more participants in addition to respiratory sounds collected in a variety of settings (e.g., competing speech), which would then be used to verify the findings reported in this work. In addition, secondary class label verification by a physician is planned for all current and future respiratory sounds. Finally, an investigation into the effect of competing audio sources (e.g., multiple residents) and/or the addition of reverberation effects may be considered.

## Supplementary Materials

In the supplemental materials available online, we have provided a discussion of some the issues with using audio visualizations as an input to image-based classifiers, a few comments on the generation of wavelet scalograms, a detailed description of the statistical metrics used in this paper, and an investigation into the impact of overlap choice when generating audio visualizations on classification performance. Finally, a description of the experiment used to determine that the oversampled dataset outperformed the unequal or undersampled datasets is included.

Supplementary materials
