# Supplementary material for: Evaluation of Respiratory Sounds Using Image-Based Approaches for Health Measurement Applications
Source: IEEE Open J Eng Med Biol. 2022 Sep 13;3:134–41. doi: 10.1109/OJEMB.2022.3202435 (PMC9788675; doi:10.1109/OJEMB.2022.3202435)
Supplement: Supplementary materials [file supp1-3202435.pdf]

Supplementary Materials

Evaluation of Respiratory Sounds using Image-based Approaches for Health Measurement Applications

Madison Cohen-McFarlane*, Graduate Student Member, IEEE, Pengcheng Xi, Bruce Wallace, Senior Member, IEEE, Karim Habashy, Saif Huq, Rafik Goubran, Fellow, IEEE, and Frank Knoefel

I. Description of the Issues when using Audio Visualizations as an Input to Image-based Approaches

In Cohen-McFarlane *et. al.* [1], the limitations of using audio visualizations as a direct input to image classifiers were described. In particular, three main issues were identified. The first was associated with the assumption that input images are spatially invariant [1]. Audio visualizations could be considered spatially invariant only along the horizontal axis (time); however, they are not invariant along the vertical axis (frequency). In other words, a sound would still be the same if played at time zero or at time one but if a sound was shifted up (frequency increase) or down (frequency decrease), the audio content would change drastically.

The second consideration was associated with assumptions about pixel representation [1]. In standard images, one can assume that a single pixel only contains information about a single object within the image. In contrast, spectrograms and scalograms are transparent in nature, meaning that a single pixel can contain information about two or more separate audio objects. This becomes more likely when working with audio signals that contain multiple sound sources and/or environmental noise (e.g., a cough during a group conversation while music is playing).

The final consideration is associated with pixel adjacency [1]. In standard images, one can assume that adjacent pixels represent the same object with the exception of boundary regions. When working with audio visualizations, a single audio object can be spread across multiple frequencies that may or may not be adjacent to each other. Based on these issues, Cohen-McFarlane *et. al.* showed that, using linear spectrograms of classical piano tones as the input to an adapted AlexNet classifier had performed better when grouping within frequency ranges (octave or frequency blocking), compared to classifying based on amplitude content appearing at different frequency levels (same note across octaves) [1].

II. Comments on the Continuous Wavelet Transform

The CWT may be affected by boundary effects along the edges of the wavelet scalogram. Generally, wavelet scalograms are presented with a highlighted area that is potentially affected by boundary effects, as seen in Fig. A of the same wet cough measurement found in Fig. 1 (m-p) in the main text. This boundary was not included in the final visualizations, as adding this to the images does not represent the original cough measurement at all.

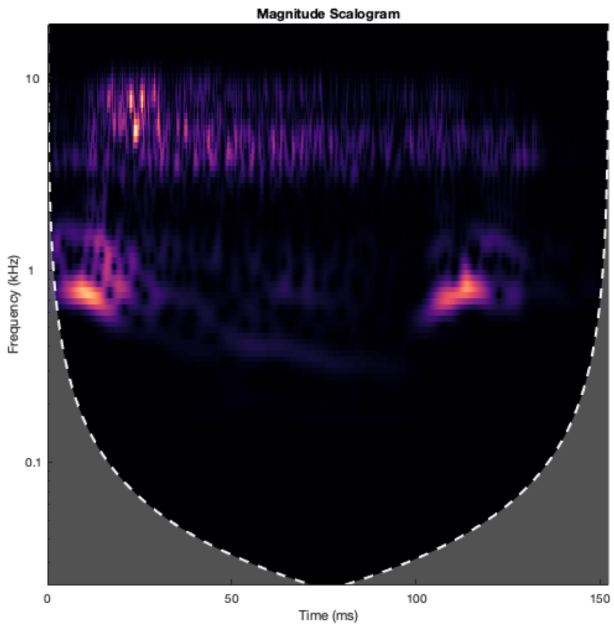

Supplementary Fig. A. Wavelet scalogram of the wet cough measurement in Fig. 1 (m-p) showing the areas of that may be affected by boundary effects.

III. Statistical Evaluation Descriptions

A. Accuracy

The accuracy for each classification task was computed based on the number of correct predictions over the total number of samples, for both the validation and testing results. When using small class sizes with fewer testing samples, validation accuracy may provide a more accurate indication of the classifier's performance. We expect that it may be the case when evaluating the performance of the undersampled dataset in some cases. Additionally, accuracy is very sensitive to class imbalance, which is present in the original dataset. Due to this, other performance measures must be considered.

B. F1-scores

The F1-score is a weighted average of precision and recall and provides a measure that highlights the balance between precision (how exact the classifier is) and recall (how complete the classifier is). The F1-score can be calculated as follows:

$$F1 = 2 * \left(\frac{P * R}{P + R}\right) \quad (1)$$

where P and R are the precision and recall of the classifier respectively.

In the case of a multiclass problem (i.e. C1), the F1-score is first computed for all classes then averaged to get an overall F1-score for the entire classifier.

C. Cohen's Kappa coefficient

The kappa coefficient reports classification accuracy that is normalized by data imbalance present in the classes. The kappa value can be computed as follows:

$$\kappa = \frac{p_o - p_e}{1 - p_e} \quad (2)$$

where p_o is the observed proportionate agreement or the standard accuracy and p_e is the overall probability that the correct class would be predicted at random. p_e is the sum of the expected probability that predictions would randomly be correct for each class.

IV. Impact of Overlap on Classification

When generating the spectrograms, 90% overlap was chosen in order to increase the pixel density in order to create visualizations that would most closely mirror standard images expected by the AlexNet classifier. Furthermore, 90% overlap is not typically used for audio classification tasks (though higher levels of overlap are used in other audio processing area including text-to-speech synthesis [2]) as higher levels of overlap may introduce correlation between adjacent frames, essentially adding redundant information.

Generally, overlap is limited to 50% when generating spectral representations. Given this two classifiers were generated, with the same training/test split following transfer learning methods described in the main manuscript, using 90% overlap and 50% overlap when generating linear spectrograms. Performance of all classification tasks across both overlapping methods are presented in Table A.

TABLE A
Linear Spectrogram Classification Results When Used as the Input to the AlexNet Transfer Learning Paradigm Using 90% and 50% Overlap

Spectrogram Overlap (%)	Task	Validation Accuracy	Testing Accuracy	Testing F1-Score	Testing Cohen's Kappa
90	C1	0.84	0.76	0.69	0.59
	C2	0.83	1.00	1.00	1.00
	C3	0.91	0.94	0.88	0.76
50	C1	0.82	0.76	0.68	0.59
	C2	0.75	1.00	1.00	1.00
	C3	0.91	0.94	0.88	0.76

The classification results are very similar across all tasks, especially when looking at the testing results in Table A. Given the similarity, 90% overlap was chosen for all further evaluations as it creates visualizations that appear to have higher resolution, more closely mimicking standard images. However, given the similarity, 50% overlap may be a more appropriate choice when implementing these methods in real-time given the reduced computational requirements with smaller overlap lengths.

V. Data Sampling Evaluation

In addition to the original unequal dataset, two other datasets were evaluated. The first addition (undersampled) semi-randomly chooses *n* samples of each class, where *n* is the number of samples in the smallest class (*n* = 19 in the dry cough dataset). Semi-random selection was chosen so that respiration sounds from different individuals were given priority over sounds from the same individual. For example, when selecting two wet coughs given a group of three sounds (P1 with two coughs and P2 with one cough), a cough from P1 and one from P2 would be selected rather than selecting both coughs from P1. This dataset was then split into separate training and testing sets as described below.

The second dataset (oversampled) focused on simplistic data augmentation methods, specifically image duplication with a noise overlay. To ensure that the testing only used held out independent data, eight samples (approximately 10% of the largest class size) from each class were set aside prior to the application of data augmentation. The remaining samples from each class (wet cough, n = 19; dry cough, n = 11; whooping cough, n = 89; restricted breathing, n = 17) were then randomly duplicated to match the largest data class (whooping cough, n = 89) and a noise overlay was applied on each duplicated image, using the '`imnoise`' function in MATLAB with varied levels of mean and variance in increments of 0.005. This method led a training dataset of unique images where each class contained the largest original number of samples (89) after removing the testing samples.

Testing samples were held out for each dataset. For the first two datasets (original and undersampled) and for each task, the data were split into 90% training and 10% testing. For the oversampled dataset, the testing samples were held out prior to data augmentation. The training data for all datasets was further divided into 70% training and 30% validation during classifier training. The AlexNex was adapted as described in the main manuscript with the following adjustments for each data sampling approach.

The minimum batch size and maximum number of epochs was chosen based on trial and error. For the original dataset, the mini batch size was set to five with a maximum of 50 epochs, consistent with the previous experiment [1]. For the undersampled dataset, the mini batch size was set to three with a maximum of 10 epochs. Finally, for the oversampled dataset, the mini batch size was set to 15 with a maximum of 10 epochs. For all tasks, the initial learning rate was set to 1.0e-4 and data was shuffled for every new epoch.

As mentioned, three different data sampling methods were considered in this work. To reduce the number of generated classifiers, the most effective data sampling method was identified using the linear spectrogram visualization method. As shown in Table B, the oversampled case has the best performance for C1, the four-class task, with a testing accuracy, F1-score, and Cohen's Kappa of 0.78, 0.78, 0.71 respectively. This is consistent with the expectation that more data can improve performance and data augmentation by adding noise empirically proves so.

We note that the original dataset has high performance for the second and third classification tasks. This may be caused by the small testing set in some of the classes consisting of only

TABLE B
LINEAR SPECTROGRAM CLASSIFICATION RESULTS WHEN USED AS THE INPUT TO THE ALEXNET TRANSFER LEARNING PARADIGM FOR ORIGINAL, UNDER-SAMPLED AND OVERSAMPLED DATASETS

Data Sampling Type	Task	Validation	Testing		
		Accuracy	Accuracy	F1-Score	Cohen's Kappa
Original	C1	0.84	0.76	0.69	0.59
	C2	0.83	1.00	1.00	1.00
	C3	0.91	0.94	0.88	0.76
Undersampled	C1	0.75	0.75	0.74	0.67
	C2	0.90	0.75	0.73	0.50
	C3	0.85	0.75	0.67	0.33
Oversampled	C1	0.97	0.78	0.78	0.71
	C2	1.00	0.81	0.81	0.63
	C3	0.97	0.91	0.86	0.71

two samples (rather than the eight testing samples used in the oversampled case). Additionally the C2 and C3 tasks are binary classification tasks, which may also falsely increase performance results observed here. Given this, we expect that the oversampled dataset presents a more realistic representation of the performance that can be achieved when evaluating independent respiratory sounds.

REFERENCES

[1] M. Cohen-McFarlane, R. Goubran, and B. Wallace, "Challenges with Audio Classification using Image Based Approaches for Health Measurement Applications," in *2020 IEEE International Symposium on Medical Measurements and Applications (MeMeA)*, Jun. 2020, pp. 1–5, doi: 10.1109/MeMeA49120.2020.9137254.

[2] T. Kaneko, K. Tanaka, H. Kameoka, and S. Seki, "iSTFTNet: Fast and Lightweight Mel-Spectrogram Vocoder Incorporating Inverse Short-Time Fourier Transform," Mar. 2022, doi: 10.48550/arxiv.2203.02395.
